# User fees in private non-for-profit hospitals in Uganda: a survey and intervention for equity

**DOI:** 10.1186/1475-9276-4-6

**Published:** 2005-05-04

**Authors:** Joseph Amone, Salome Asio, Adriano Cattaneo, Annet Kakinda Kweyatulira, Anna Macaluso, Gavino Maciocco, Maurice Mukokoma, Luca Ronfani, Stefano Santini

**Affiliations:** 1 Uganda Catholic Medical Bureau, Kampala, Uganda; 2 Ugandan Martyrs University, Nkozi, Uganda; 3 Unit for Health Services Research and International Health, IRCCS Burlo Garofolo, Via dell'Istria 65/1, 34137 Trieste, Italy; 4 Cuamm – Doctors with Africa, Padova, Italy

## Abstract

**Background:**

In developing countries, user fees may represent an important source of revenues for private-non-for-profit hospitals, but they may also affect access, use and equity.

**Methods:**

This survey was conducted in ten hospitals of the Uganda Catholic Medical Bureau to assess differences in user fees policies and to propose changes that would better fit with the social concern explicitly pursued by the Bureau. Through a review of relevant hospital documents and reports, and through interviews with key informants, health workers and users, hospital and non-hospital cost was calculated, as well as overall expenditure and revenues. Lower fees were applied in some pilot hospitals after the survey.

**Results:**

The percentage of revenues from user fees varied between 6% and 89% (average 40%). Some hospitals were more successful than others in getting external aid and government subsidies. These hospitals were applying lower fees and flat rates, and were offering free essential services to encourage access, as opposed to the fee-for-service policies implemented in less successful hospitals. The wide variation in user fees among hospitals was not justified by differences in case mix. None of the hospitals had a policy for exemption of the poor; the few users that actually got exempted were not really poor. To pay hospital and non-hospital expenses, about one third of users had to borrow money or sell goods and property. The fee system applied after the survey, based on flat and lower rates, brought about an increase in access and use of hospital services.

**Conclusion:**

Our results confirm that user fees represent an unfair mechanism of financing for health services because they exclude the poor and the sick. To mitigate this effect, flat rates and lower fees for the most vulnerable users were introduced to replace the fee-for-service system in some hospitals after the survey. The results are encouraging: hospital use, especially for pregnancy, childbirth and childhood illness, increased immediately, with no detrimental effect on overall revenues. A more equitable user fees system is possible.

## Background

Since their institution, user fees have been used in private non-for-profit (PNFP) hospitals as a way to finance provision of services. The level of cost recovery, as a percentage of recurrent expenditure, is low in government health services [1], but is thought to contribute an average of 50–60% of PNFP hospital revenues and about 90–100% of the revenues of lower level health facilities in Uganda [2]. Yet, very little is known about the structure of user fees, their predictability for users, their effects on the use of PNFP health services, and the levels of payers' compliance. In the absence of other options of community financing, such as pre-payment schemes, user fees are likely to remain the main source of financing for the provision of health services in the PNFP health sector in Uganda and other sub-Saharan countries. But the PNFP health sector operates out of social concern and explicitly pursues equity and accessibility for the poor [3]. It is therefore of absolute importance to assess current practices, their effect on patterns and trends of use, and the additional suffering all this may add on patients, in order to address and correct inequities. The aim of this study was to offer a tool for a more focused and rational structure and management of user fees in PFNP health facilities belonging to the Uganda Catholic Medical Bureau (UCMB), in an overall health system perspective (i.e. coherently with government policies) and with equity of access in mind.

## Methods

**Figure 1:**
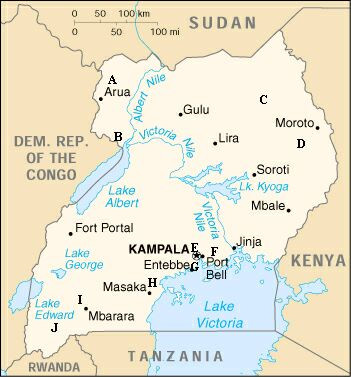
The location (A to J) of the ten hospitals.

The survey was conducted in a convenient sample of ten hospitals (Figure 1), chosen by UCMB using the following criteria: availability of relevant documents, geographical distribution, variety of user fee policies, willingness to support the study and to use its results for policy changes. Seven surveyors in two teams gathered data during site visits in five weeks (one week per hospital between 3 July and 13 August 2000). The surveyors gathered and reviewed hospital documents (annual and financial reports for the previous five years; logbooks from different departments, including accountants' books; planning, budgeting and other financial documents, including agreements with the Ministry of Health; price lists for drugs and other services), and interviewed managers (group interviews with medical superintendents, senior nursing officers, hospital administrators, and accountants), health workers (individual interviews with professionals in charge of outpatients, inpatients, pharmacy, laboratory, X-rays, and preventive services), and users (30 outpatients and 10 inpatients per hospital). Outpatients were supposed to be selected through systematic sampling and stratified by age and sex (ten children under 10 years, ten adult women, and ten adult men). In some hospitals, however, the number of outpatients was so low at the time of the visit that all those available were consecutively interviewed trying to maintain the proposed distribution by age and sex. Inpatients were selected among those being discharged during the visit from maternity, children and adult wards. The questionnaires had been developed in advance and were field tested and slightly modified during the first hospital visit. The interviews with users were conducted by the local surveyors in the local language.

Cost is reported in US dollars ($). The exchange rate at the time of the survey was 1 $ = 1,600 Ugandan shillings. In the user survey, cost includes hospital fees and other cost that users had to meet to get health care (non-hospital cost), such as travel expenses, indirect cost (money lost because of lost work), and money already spent for the same episode of illness in other health services, shops or pharmacies. The total cost is the amount paid by users for the whole episode of illness. Total cost, however, might be underestimated due to lack of information, and because of exemptions and/or inability to pay. Hospital fees were calculated from hospital receipts, while users estimated non-hospital cost.

The preliminary results of the survey were used to identify a group of hospitals where an intervention aimed at ensuring an equitable access was considered a priority. The main intervention consisted in the progressive replacement of the fee-for-service system with a system based on flat fees covering all the services provided for a single episode of disease, at both inpatient and outpatient levels. The flat fees for maternal and child health care and for some long term conditions were on average lowered more than the fees for other conditions. The fees previously charged for preventive services (e.g., antenatal care) were abolished or reduced to a token initial payment. The public was informed of these changes in fees level and system. Simultaneously, the system for accounting and financial reporting was streamlined and made transparent; health workers got a higher and more regular salary, including all the social benefits envisaged by the Ugandan law, as an incentive to improved quality of care [4]; the acquisition, storage and use of drugs and consumables was strictly monitored and rationalised.

## Results

The catchment population of the ten hospitals ranged between 53,000 and 172,000; the number of beds from 92 to 320. The number of doctors varied from 3 to 28, that of qualified nurses and midwives from 16 to 115. Small hospitals tended to have a higher rate of hospital staff per 100 beds compared to larger hospitals, except for the large hospital E in Kampala, which had by far the highest rate. Table 1 shows some measures of hospital activity in 1999.

**Table 1:** Some measures of hospital activity (1999).

Hospital	Total admissions	Deliveries	Cesarean section (%)	Length of stay (days)	Bed occupancy (%)	Outpatient visits	Antenatal visits
A	4,234	378	28	17.0	85	8,303	3,061
B	7,809	1,232	18	10.5	93	14,793	6,527
C	8,825	1,824	6	11.4	86	44,791	10,883
D	7,730	548	15	9.9	95	34,509	1,509
E	8,193	2,031	16	6.7	50	76,303	9,508
F	2,531	544	26	5.1	23	6,139	2,071
G	2,143	378	12	5.9	38	9,896	2,674
H	1,498	131	29	5.2	21	17,379	1,551
I	4,419	943	26	8.8	61	11,189	3,388
J	5,722	326	36	NA	NA	13,984	1,387

The annual hospital expenditure ranged between $ 72,000 (F) and 1.07 million (E) in the financial year 1998/99 (mean $ 288,750), the last for which reports were available from all hospitals; but excluding hospital E the upper limit dropped to about $ 451,000 (D). Employment cost represented the largest portion of expenditure: from 27% (H) to 47% (F) (mean 35%); followed by medical goods and services: 7% (C) to 26% (J) (mean 16%). Table 2 shows the revenues for the same financial year for each hospital, and its sources grouped into user fees (outpatients and inpatients), government subsidies (the financial support given by the Ministry of Health to each hospital based on a service level agreement), external aid (only financial), and other sources (including donation in kind and services, income generating activities, credits, savings and interest on savings). The percentage from user fees varied widely among hospitals, from 6% to 89%, but on average reached 40%, slightly less than previously thought [2]. The difference between northern and southern hospitals was clear-cut. It was also clear that some hospitals were more successful than others in getting external aid and government subsidies.

**Table 2:** Source of revenues in the financial year 1998/99.

Hospital	Total revenues ($)	Percentage from:				
		
		User fees (outpatients)	User fees (inpatients)	Government subsidies	External aid	Other sources
A	187,000	8	11	18	40	22
B	260,000	6	12	16	18	48
C	355,000	3	3	8	70	17
D	584,000	6	5	14	23	52
E	1,183,000	27	38	1	25	8
F	72,000	17	57	25	0	1
G	112,000	39	50	3	6	3
H	143,000	14	15	26	32	14
I	218,000	9	60	12	0	19
J	145,000	23	55	18	0	3
Average	349,000	15	25	10	28	22

From hospital records and reports, the average outpatient fee appeared to vary between $ 0.28 (C) and 4.38 (G) (mean $ 1.75); for inpatients, the average fee was between $ 1.56 (C) and 55.38 (E) (mean $ 16.02). Most hospitals adopted a fee-for-service system, and very often users had to pay even for preventive services such as antenatal care. The typical outpatient bill included consultation (with a nurse or a clinical officer), drugs, lab tests and X-rays, if any; consultation with a doctor meant an extra charge. Inpatients, in addition to the above, had to pay for hospital stay (general expenses, bed stay, doctor fee), for supplies consumed and for the surgery, if any. In some northern hospitals, a set of services was subsidised (free, low cost or flat rates): care for common childhood illness, pregnancy and childbirth, and some common medical and obstetric conditions. In all hospitals, drugs for tuberculosis, STI and HIV/AIDS were subsidised by the government and administered free to patients; but in most hospitals these patients had to pay for other services. Most patients with chronic conditions had to pay the full fee. Only in one hospital (D) was the fee system adjusted to favour a more rational use of the referral system in the district and sub-district.

**Figure 2:**
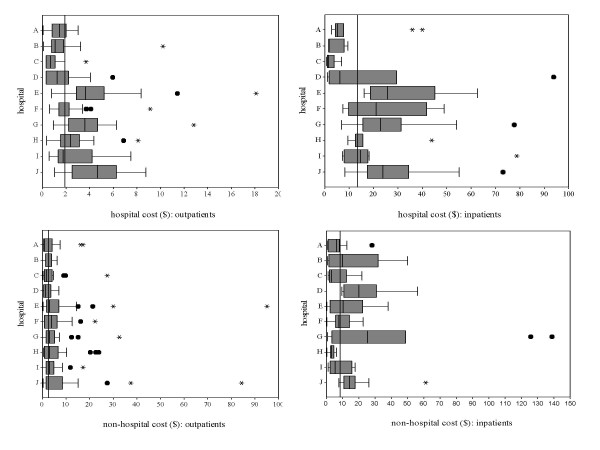
**Hospital and non-hospital cost borne by outpatients and inpatients. **The limits of each box represent the 25^th ^and 75^th ^percentile values; whiskers represent the highest and lowest values excluding outliers; extreme values are shown as dots (1.5 to 3 times the box length) and asterisks (3 times or more the box length); vertical lines represent the median value in each hospital and overall.

Figure 2 shows hospital, i.e. the fees the 303 outpatients and the 102 inpatients interviewed actually paid, and non-hospital cost, i.e. the amount of money patients declared that they had paid for travel and for seeking care elsewhere for the same episode of illness, plus their estimated loss of income due to illness and careseeking. The patients were similar in age and sex distribution in all hospitals. They were coming from a mean distance of 12 (outpatients) and 23 km (inpatients); about 45% were living within 5 km. Education was overall higher for men than for women, with 16% of illiteracy among men and 31% among women. Women were also more unemployed than men: 79% vs. 45%. Non-hospital cost was much less variable among sites than hospital cost, and was proportionally higher among outpatients than among inpatients. Total cost can be calculated by the sum of hospital and non-hospital cost.

**Figure 3:**
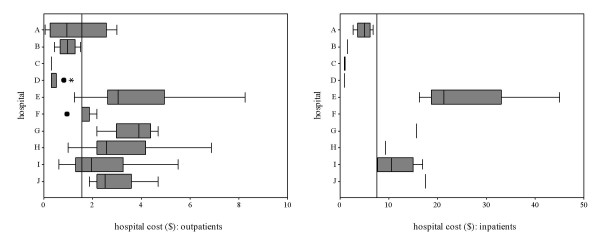
**Hospital cost borne by outpatient and inpatient children (less than 15 years) with malaria. **The limits of each box represent the 25^th ^and 75^th ^percentile values; whiskers represent the highest and lowest values excluding outliers; extreme values are shown as dots (1.5 to 3 times the box length) and asterisks (3 times or more the box length); vertical lines represent the median value in each hospital and overall; absence of box and whiskers means less than three cases; the value for hospital F (inpatients) coincides with the overall median.

The wide variation in user fees among hospitals was not justified by differences in case mix: most patients were seen or admitted for a very small range of conditions (malaria, diarrhoea, respiratory infections, accidents, normal or complicated delivery) in all hospitals. The variation remained even when user fees were compared for the same diagnosis among hospitals. Figure 3 shows the example of malaria, the most common diagnosis among all patients interviewed, in children aged less than 15 years seen as outpatients or admitted as inpatients. A child with malaria paid 1 US $ for admission in hospitals C and D, and 20 times that amount in hospital E. A severity score would help interpreting these figures, but it is difficult to imagine a difference in severity that would completely explain this difference in cost. The same variation was observed for other common conditions.

At the time of the interview, 220 outpatients (73%) had fully paid their fees, 36 (12%) had paid partially, and 47 (16%) had not paid; among these, 22 were treated on credit and 21 were exempted; the non-payers were only four. It was impossible to interview all the inpatients after payment; some were interviewed the day before discharge or as soon as discharge was decided. Some patients referred that in previous episodes of illness they were denied or reduced treatment, drugs in particular, if they could not afford to pay. Few hospitals held funds to help the poor; according to hospital managers, poor patients would be exempted after some form of subjective judgement by members of the hospital management team. For this reason, indigent patients tended to avoid these hospitals in favour of other facilities, mostly government services. Out of 303 outpatients, only 34 (11%) benefited from some kind of exemption; among them, 11 were part of the hospital staff and the others were teachers or pupils of the catholic school, parish clerks and soldiers. Only one outpatient was exempted because he was poor. The inpatients who benefited from some kind of exemption were 6 out of 102 (6%): five for specific health conditions and one for indigence.

To pay hospital and non-hospital expenses, 34% of outpatients and 42% of inpatients had to borrow money; 24% and 30%, respectively, had to sell goods and property. Moneylenders were usually members of the family; interestingly, 22 women said that they had to borrow money from their husband. Regarding the sale of goods, outpatients sold vegetables (19/67, 28%), cash crops (11/67, 16%), or animals (20/67, 30%); among inpatients, 50% (16/32) sold animals and 13% (4/32) part of their land. Patients found particularly difficult to afford hospital fees during the planting season and at the time of enrolling children at school. Lack of money was the most important reason for seeking care elsewhere for the same episode of illness (more than 50% of users) or for coming late for care (38%).

**Figure 4:**
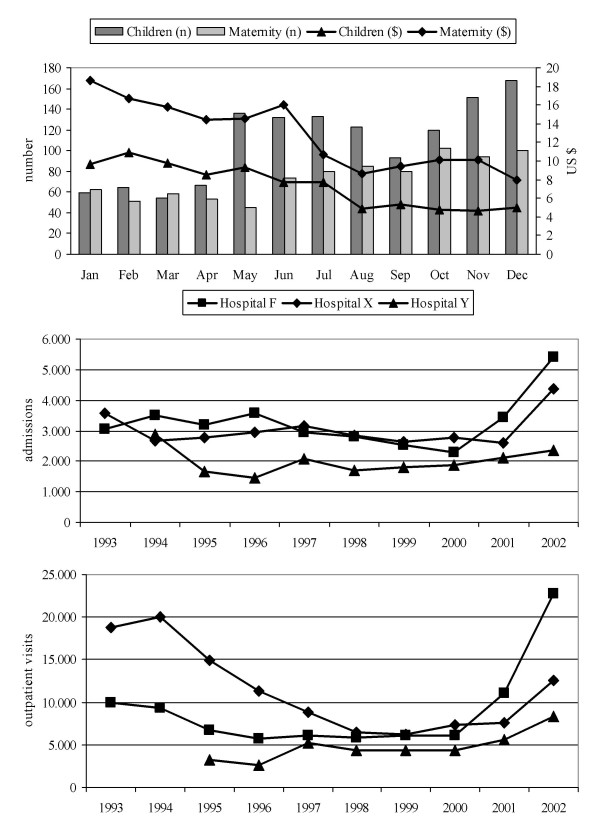
**Selected measures of hospital activity before and after the intervention. **Number of admissions and average fees in the children and maternity wards of hospital F in 2001 (above), and number of admissions and outpatient visits per year between 1993 and 2002 in hospitals F, X and Y (below).

From March 2001, the intervention was progressively applied in hospital F and in nearby hospital X, while hospital Y served as control (modest reduction of fees only for outpatients). Hospitals X and Y were not included in the survey and belong with hospital F to the same diocesis. Figure 4 shows the number of admissions and average fees in the children and maternity wards in 2001 in hospital F; it shows also the number of admissions and outpatient visits per year in hospitals F, X and Y between 1993, when user fees were widely introduced, and 2002. The upward trend after lowering the fees is clear. In hospital F the number of deliveries increased from 456 in 2000 to 605 in 2001, with 136 and 116 caesarean sections, respectively. Bed occupancy went up from 23% to 46% in the same period (up to 63% in December 2001), mostly because of the 185% and 63% increase in admissions in children and maternity wards. The reduction of average user fees was compensated by increased use, and the monthly revenue from user fees actually increased, from $ 2693 in January to $ 3421 in December 2001. The revenue for user fees was $ 33,630 in 2000 and $ 35,050 in 2001; due to the simultaneous growth of government subsidies, from $ 12,690 to $ 37,280, granted because of better access and use, the proportion of revenue from user fees went down from 73% to 41%. Despite an increased expenditure for salaries, the financial statement for 2001 showed a positive balance.

## Discussion

The results of this study can not be generalised because it was not carried out on a random sample of hospitals and users. But the information on fees appears to be reliable because the average fee per patient calculated from annual reports and financial statements is similar to the median fee estimated through interviews. Our results show wide differences among hospitals; these differences do not seem to reflect social and economic differences of users, except perhaps for hospital E in Kampala, nor differences in terms of offered services. Our results also suggest that the level and structure of user fees may affect access and use of services, as shown by other studies [1,5-11].

User fees are generally considered an unfair mechanism of financing for health services; they represent a barrier to access for the poor and the powerless, and they discriminate the sickest [12]. Even more so when they are not predictable by the users as in the majority of the hospitals we surveyed. Combined with the unpredictability of illness, user fees expose people to the risk of severe economic crisis from which it may prove difficult to emerge [13,14]. Exemption mechanisms are unlikely to soften these negative effects of user fees [15]. For these reasons, user fees interfere with the mission of the UCMB, which is to serve the entire population but in particular the most vulnerable groups.

But user fees represented a large proportion of hospital revenues in five of the surveyed hospitals. More equitable systems of health services financing, such as social health insurance and progressive tax contributions, must be designed but are far from being implemented in sub-Saharan Africa [16]. External aid could play an important role but its amount and regularity can not be anticipated. Government subsidies are low and unlikely to increase dramatically; they would not compensate for reduced revenue from user fees. A reduction of the already low hospital expenditure is also unlikely, although an improvement in efficiency may lead to better services provided at the same cost. As regrettable as it may sound, user fees are likely to remain an important factor for the sustainability of PNFP hospitals in Uganda and other countries in sub-Saharan Africa for many years.

## Conclusion

The fee system applied in the pilot hospitals, i.e. flat rates and lower overall fees with reduced charges for the more vulnerable groups, after the survey seems feasible and effective. It is hoped that its extension to other PNFP hospitals in Uganda and elsewhere may ensure that a proportion of annual revenues (20% to 30% on average) continue to derive from user fees without negative effects on access, use and equity. This fee system, combined with regular revenue from government subsidies and some external aid, could allow these hospitals to survive while more pro-poor public financing systems are developed and implemented.

## Competing interests

The author(s) declare that they have no competing interests.

## Authors' contributions

AC, AM and GM conceived and designed the study. SS organised the field survey and was in charge of the post-survey intervention. JA, SA, AC, AKK, AM, GM and MM conducted the field survey and gathered all the data, except those gathered by SS during the post-survey intervention. LR, AM, AC, GM and SS analysed the data; the interpretation was shared with the whole team. GM and AM wrote the initial report based on which AC drafted and finalised the paper. All authors read and approved the final manuscript.
